# Supplementary figures and images for: TIPE drives a cancer stem-like phenotype by promoting glycolysis via PKM2/HIF-1α axis in melanoma
Source: eLife. 2024 Dec 27;13:RP92741. doi: 10.7554/eLife.92741 (PMC11677236; doi:10.7554/eLife.92741)

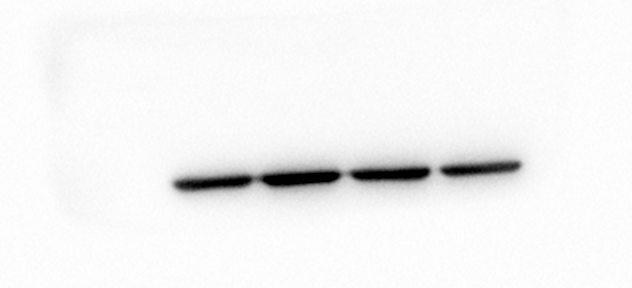

Supplement: Figure 1—figure supplement 1—source data 1. [file elife-92741-fig1-figsupp1-data1.zip › Figure 1-figure supplement 1-source data 2/1a actin.tif]

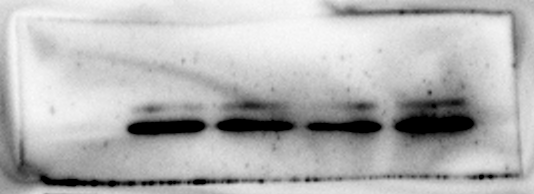

Supplement: Figure 1—figure supplement 1—source data 1. [file elife-92741-fig1-figsupp1-data1.zip › Figure 1-figure supplement 1-source data 2/1a TIPE.tif]

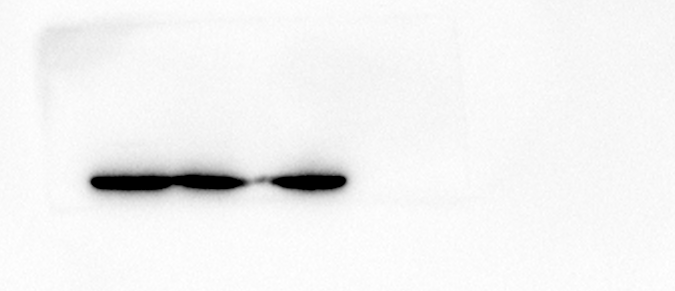

Supplement: Figure 1—figure supplement 1—source data 1. [file elife-92741-fig1-figsupp1-data1.zip › Figure 1-figure supplement 1-source data 2/1b actin.tif]

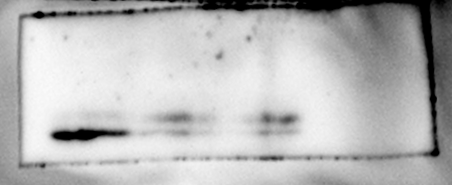

Supplement: Figure 1—figure supplement 1—source data 1. [file elife-92741-fig1-figsupp1-data1.zip › Figure 1-figure supplement 1-source data 2/1b TIPE.tif]

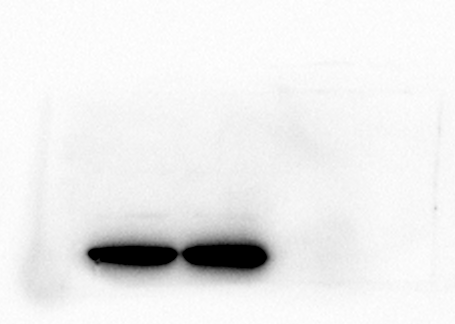

Supplement: Figure 1—figure supplement 1—source data 1. [file elife-92741-fig1-figsupp1-data1.zip › Figure 1-figure supplement 1-source data 2/1c actin.tif]

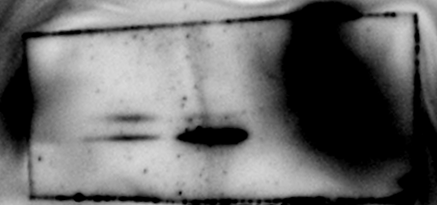

Supplement: Figure 1—figure supplement 1—source data 1. [file elife-92741-fig1-figsupp1-data1.zip › Figure 1-figure supplement 1-source data 2/1c TIPE.tif]

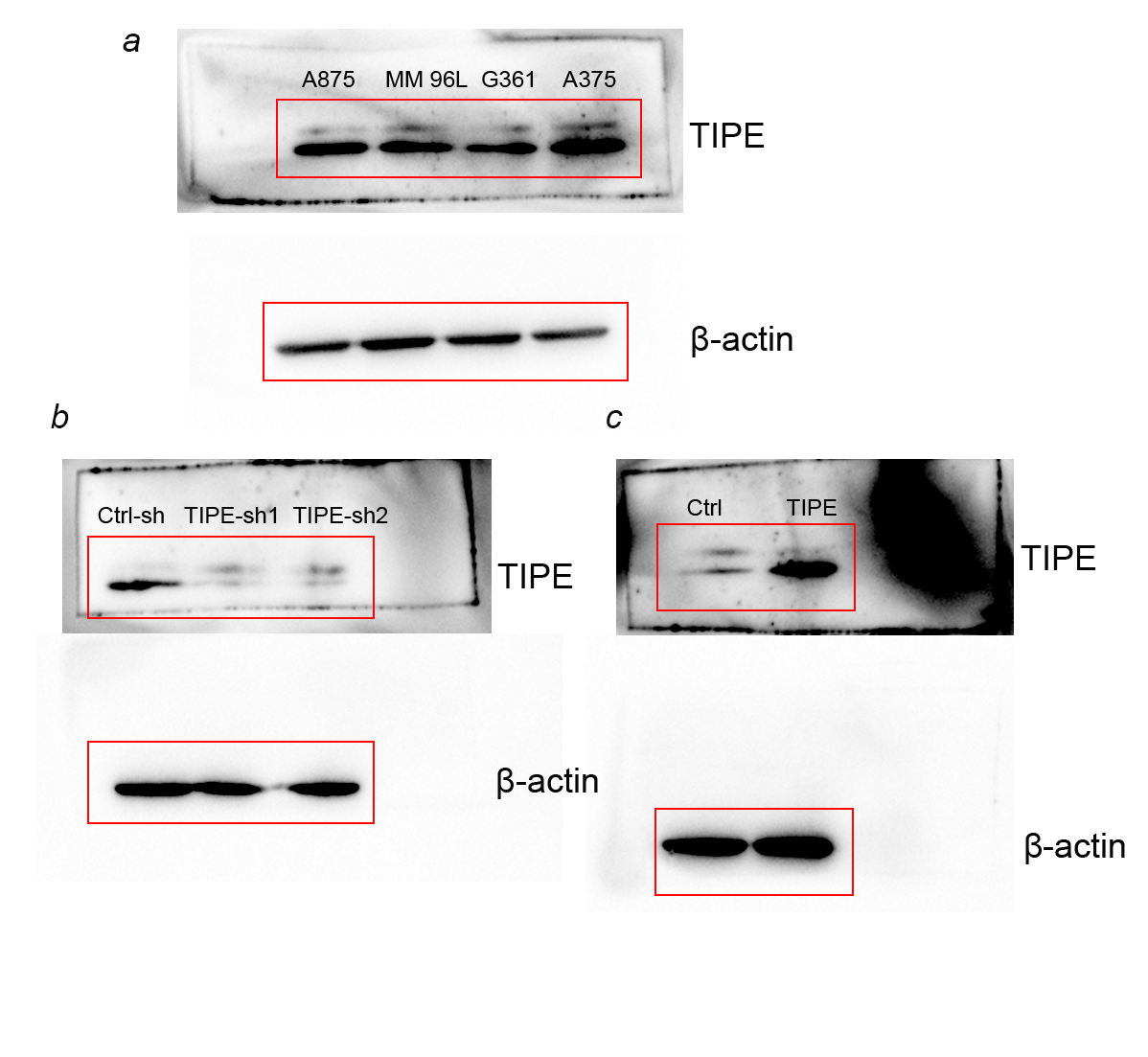

Supplement: Figure 1—figure supplement 1—source data 2. [file elife-92741-fig1-figsupp1-data2.zip › Figure 1-figure supplement 1-source data 2.tif]

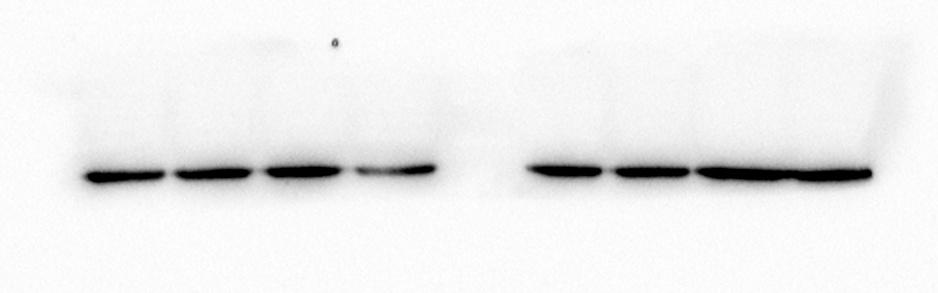

Supplement: Figure 1—figure supplement 4—source data 1. [file elife-92741-fig1-figsupp4-data1.zip › Figure 1-figure supplement 4-source data 2/4b actin.tif]

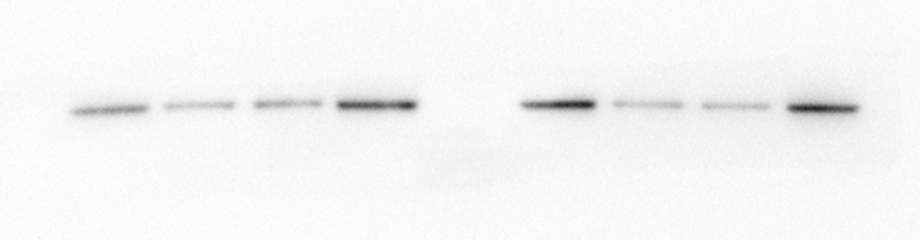

Supplement: Figure 1—figure supplement 4—source data 1. [file elife-92741-fig1-figsupp4-data1.zip › Figure 1-figure supplement 4-source data 2/4b HIF-1a.tif]

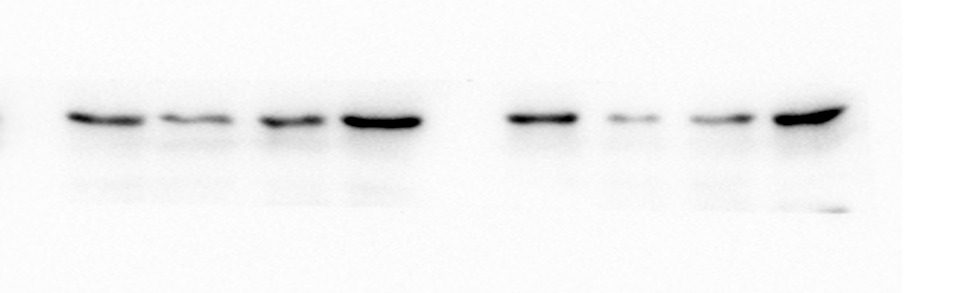

Supplement: Figure 1—figure supplement 4—source data 1. [file elife-92741-fig1-figsupp4-data1.zip › Figure 1-figure supplement 4-source data 2/4b TIPE.tif]

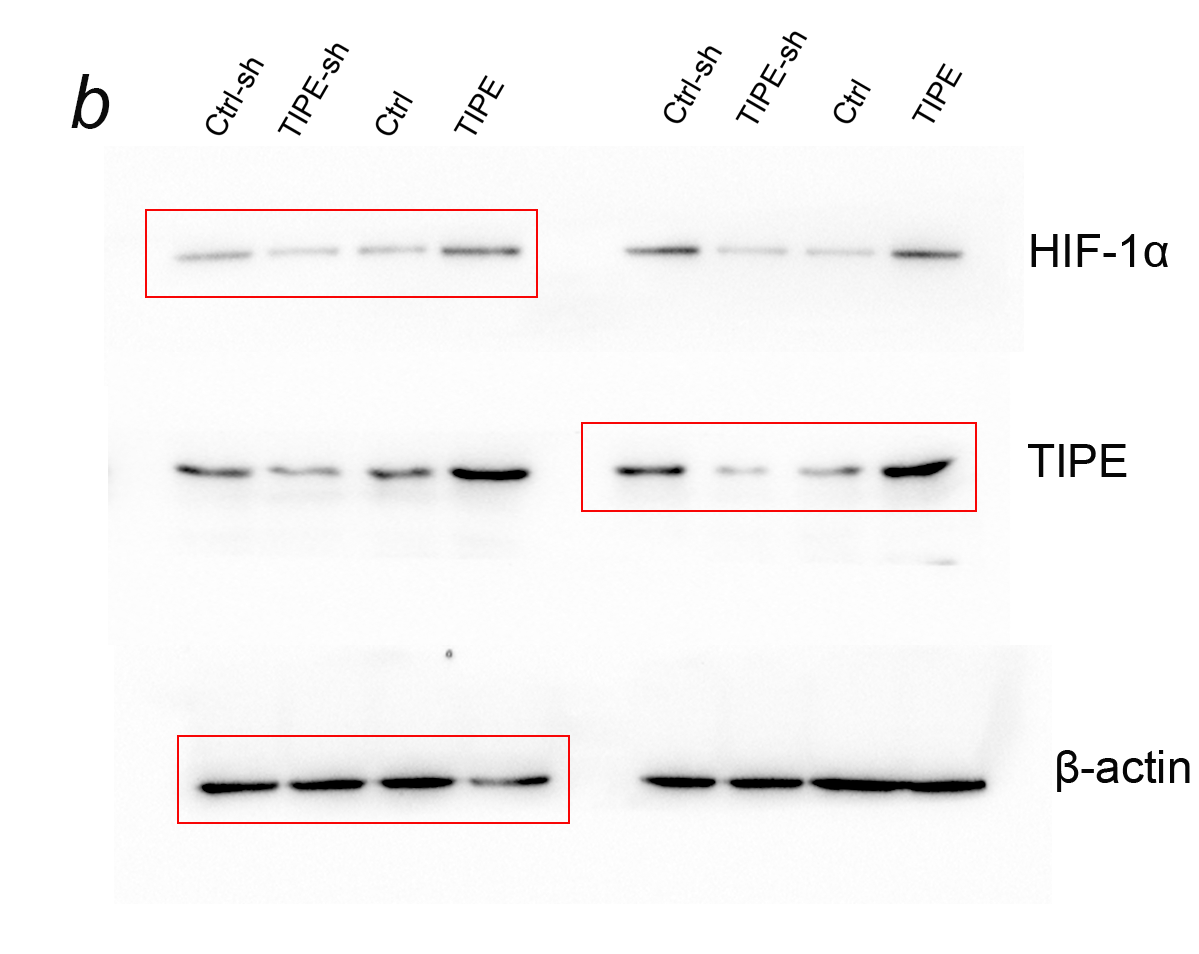

Supplement: Figure 1—figure supplement 4—source data 2. [file elife-92741-fig1-figsupp4-data2.zip › Figure 1-figure supplement 4-source data 2.tif]

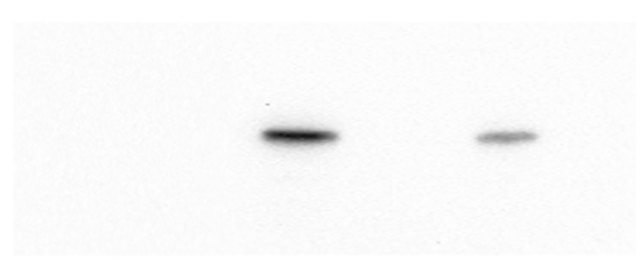

Supplement: Figure 2—source data 1. [file elife-92741-fig2-data1.zip › Figure 2-source data 2/Fig 2B PKM2-Flag.tif]

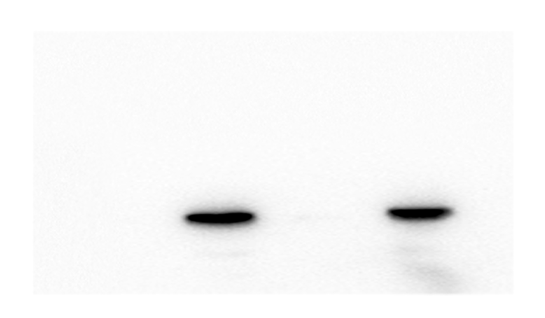

Supplement: Figure 2—source data 1. [file elife-92741-fig2-data1.zip › Figure 2-source data 2/Fig 2B TIPE-HA.tif]

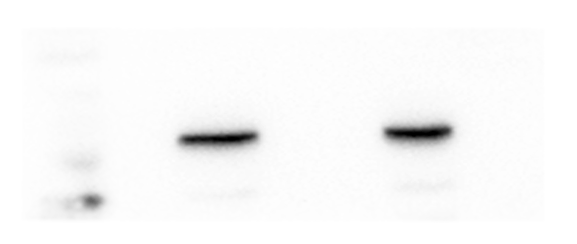

Supplement: Figure 2—source data 1. [file elife-92741-fig2-data1.zip › Figure 2-source data 2/Fig 2C PKM2-Flag.tif]

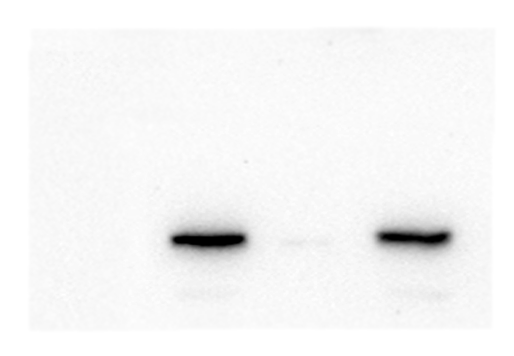

Supplement: Figure 2—source data 1. [file elife-92741-fig2-data1.zip › Figure 2-source data 2/Fig 2C TIPE-HA.tif]

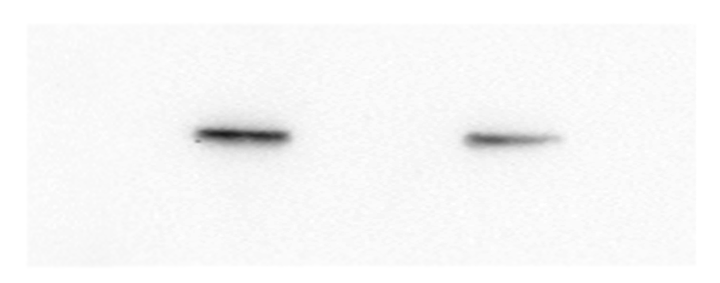

Supplement: Figure 2—source data 1. [file elife-92741-fig2-data1.zip › Figure 2-source data 2/Fig 2D PKM2.tif]

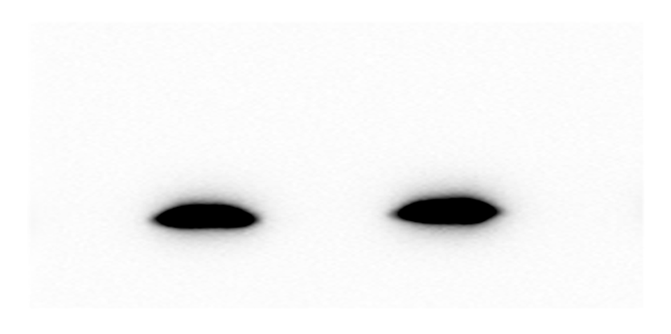

Supplement: Figure 2—source data 1. [file elife-92741-fig2-data1.zip › Figure 2-source data 2/Fig 2D TIPE.tif]

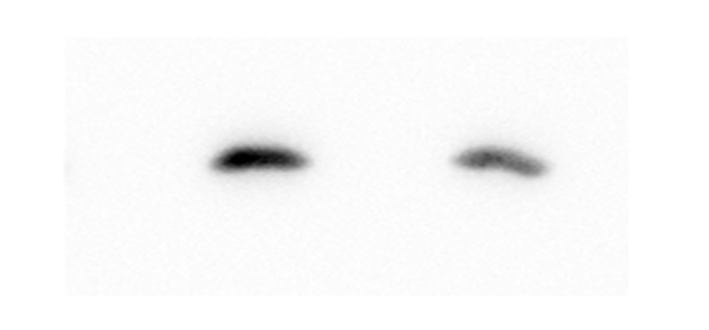

Supplement: Figure 2—source data 1. [file elife-92741-fig2-data1.zip › Figure 2-source data 2/Fig 2E PKM2.tif]

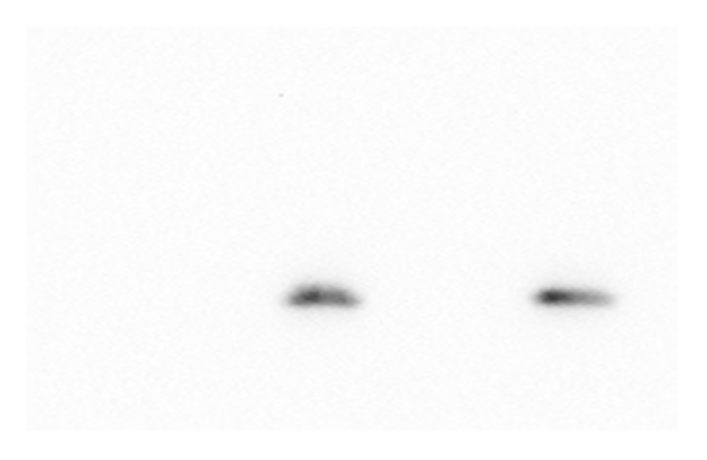

Supplement: Figure 2—source data 1. [file elife-92741-fig2-data1.zip › Figure 2-source data 2/Fig 2E TIPE.tif]

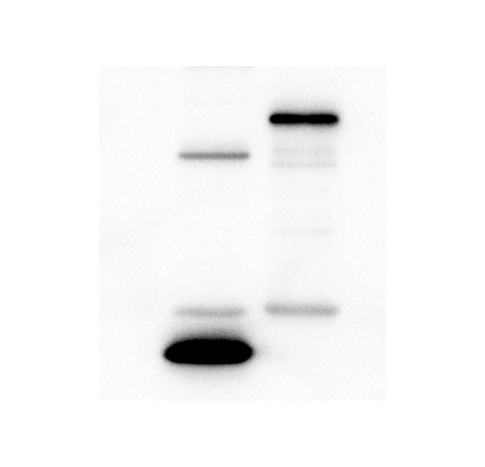

Supplement: Figure 2—source data 1. [file elife-92741-fig2-data1.zip › Figure 2-source data 2/Fig 2F GST-PKM2.tif]

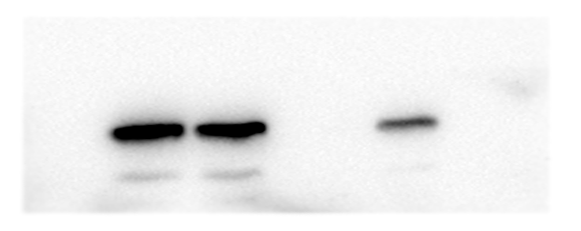

Supplement: Figure 2—source data 1. [file elife-92741-fig2-data1.zip › Figure 2-source data 2/Fig 2F TIPE-HA.tif]

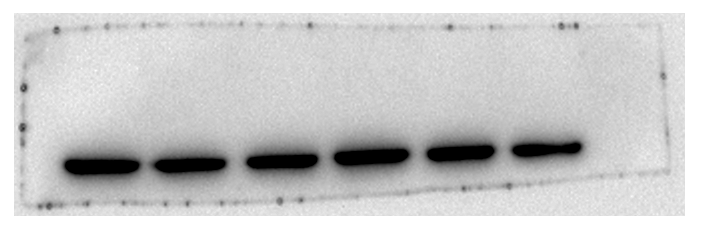

Supplement: Figure 2—source data 1. [file elife-92741-fig2-data1.zip › Figure 2-source data 2/Fig 2I GAPDH.tif]

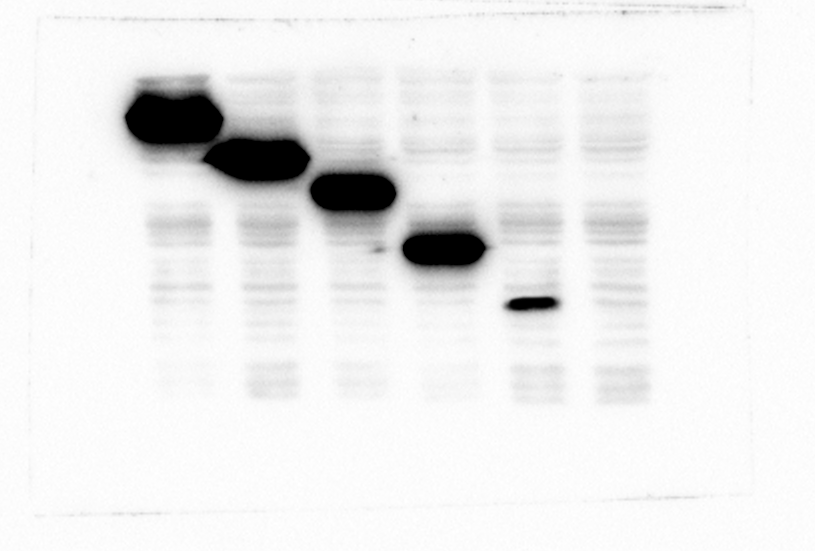

Supplement: Figure 2—source data 1. [file elife-92741-fig2-data1.zip › Figure 2-source data 2/Fig 2I Input-Flag.tif]

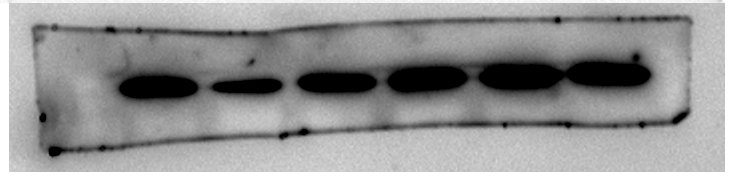

Supplement: Figure 2—source data 1. [file elife-92741-fig2-data1.zip › Figure 2-source data 2/Fig 2I Input-HA.tif]

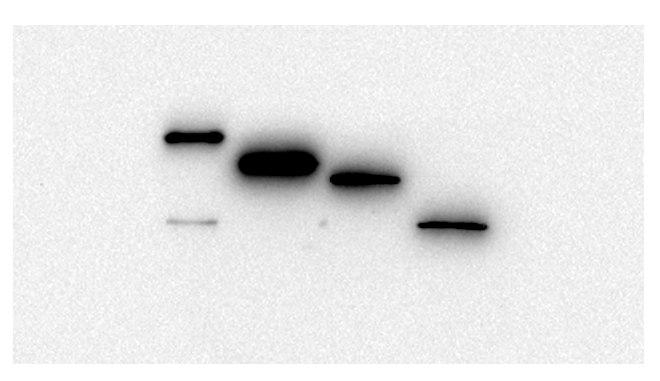

Supplement: Figure 2—source data 1. [file elife-92741-fig2-data1.zip › Figure 2-source data 2/Fig 2I IP-Flag.tif]

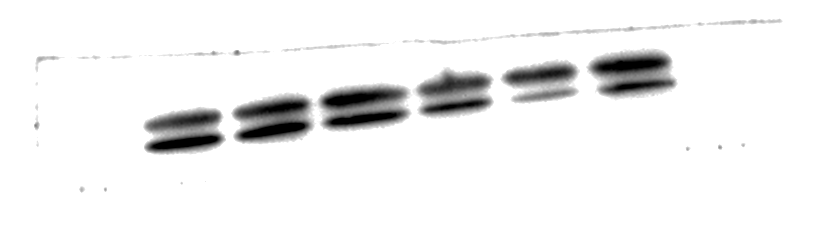

Supplement: Figure 2—source data 1. [file elife-92741-fig2-data1.zip › Figure 2-source data 2/Fig 2I IP-HA.tif]

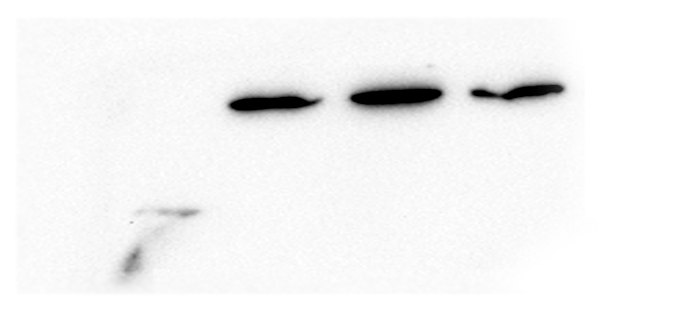

Supplement: Figure 2—source data 1. [file elife-92741-fig2-data1.zip › Figure 2-source data 2/Fig 2J Input-Flag.tif]

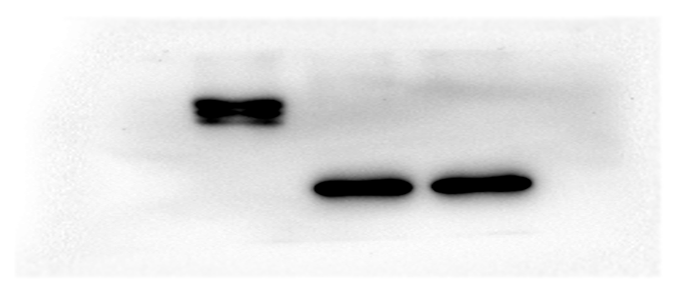

Supplement: Figure 2—source data 1. [file elife-92741-fig2-data1.zip › Figure 2-source data 2/Fig 2J Input-HA.tif]

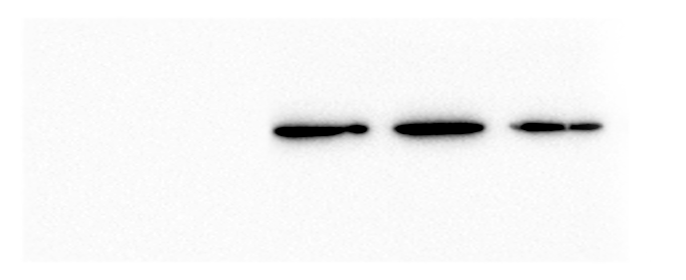

Supplement: Figure 2—source data 1. [file elife-92741-fig2-data1.zip › Figure 2-source data 2/Fig 2J IP-Flag.tif]

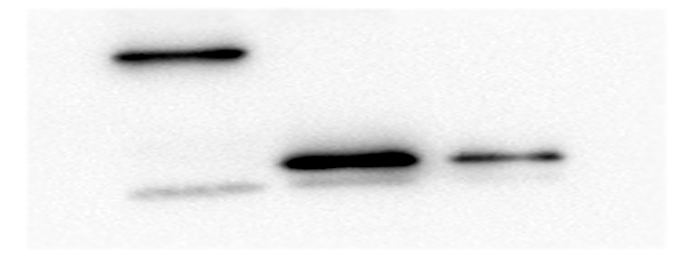

Supplement: Figure 2—source data 1. [file elife-92741-fig2-data1.zip › Figure 2-source data 2/Fig 2J IP-HA.tif]

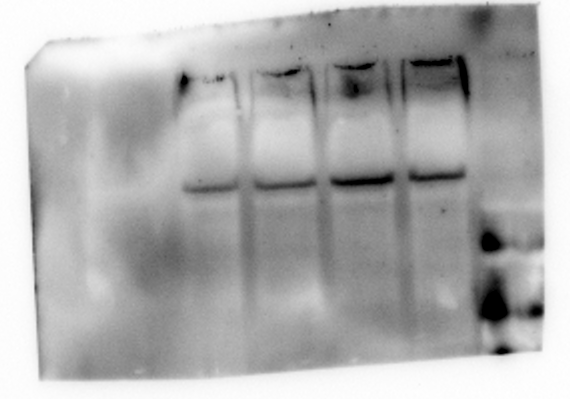

Supplement: Figure 2—source data 1. [file elife-92741-fig2-data1.zip › Figure 2-source data 2/Fig 2K.tif]

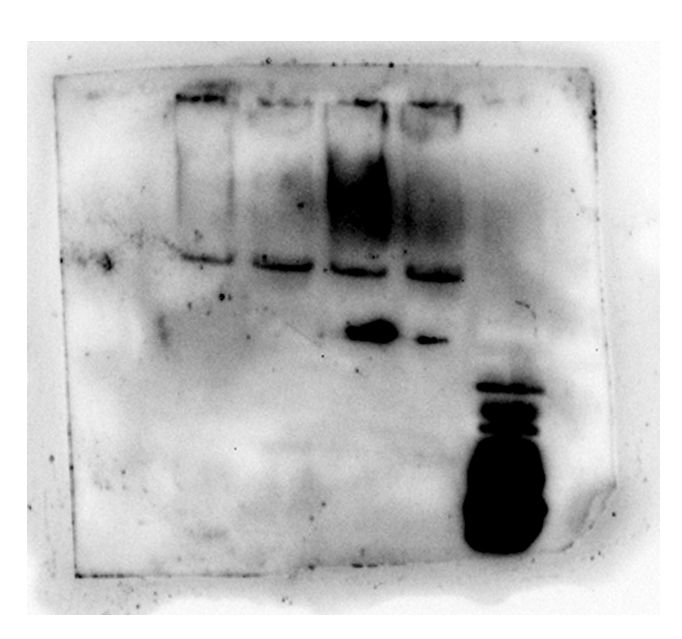

Supplement: Figure 2—source data 1. [file elife-92741-fig2-data1.zip › Figure 2-source data 2/Fig 2L.tif]

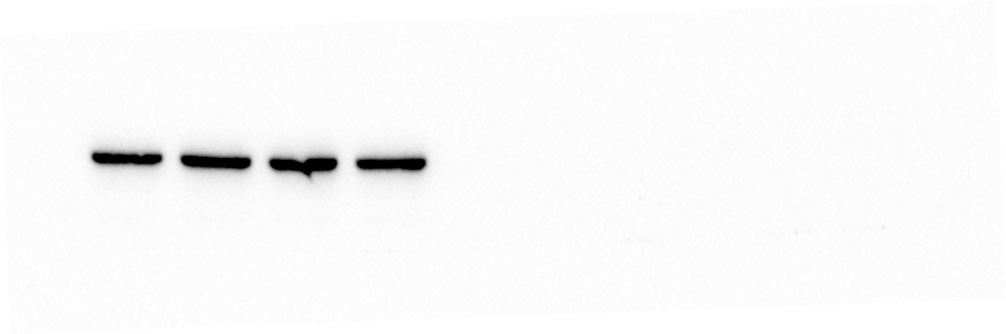

Supplement: Figure 2—source data 1. [file elife-92741-fig2-data1.zip › Figure 2-source data 2/Fig 2M-actin.tif]

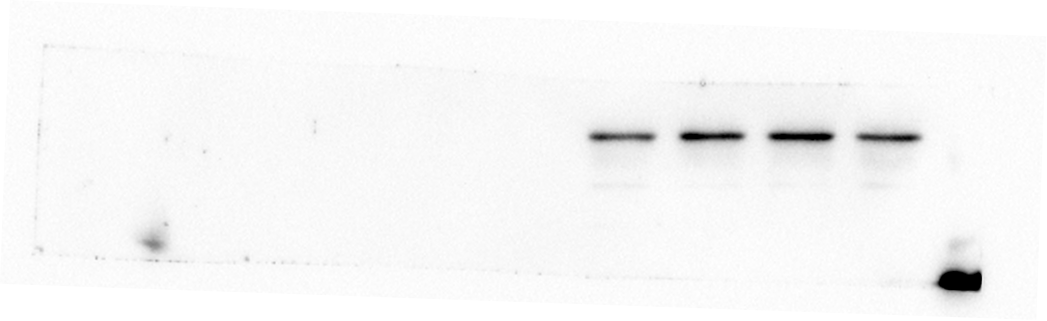

Supplement: Figure 2—source data 1. [file elife-92741-fig2-data1.zip › Figure 2-source data 2/Fig 2M-LaminB.tif]

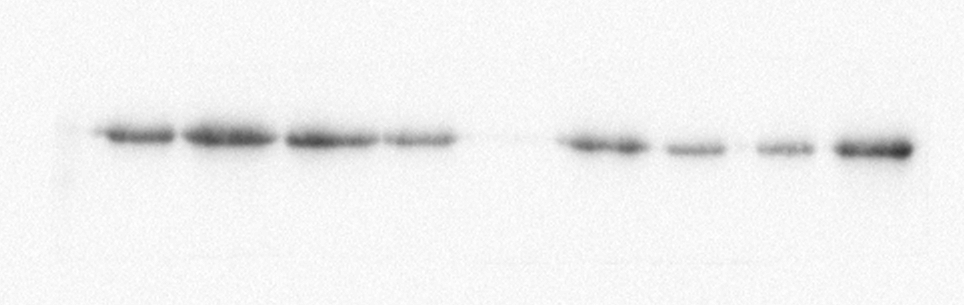

Supplement: Figure 2—source data 1. [file elife-92741-fig2-data1.zip › Figure 2-source data 2/Fig 2M-PKM2.tif]

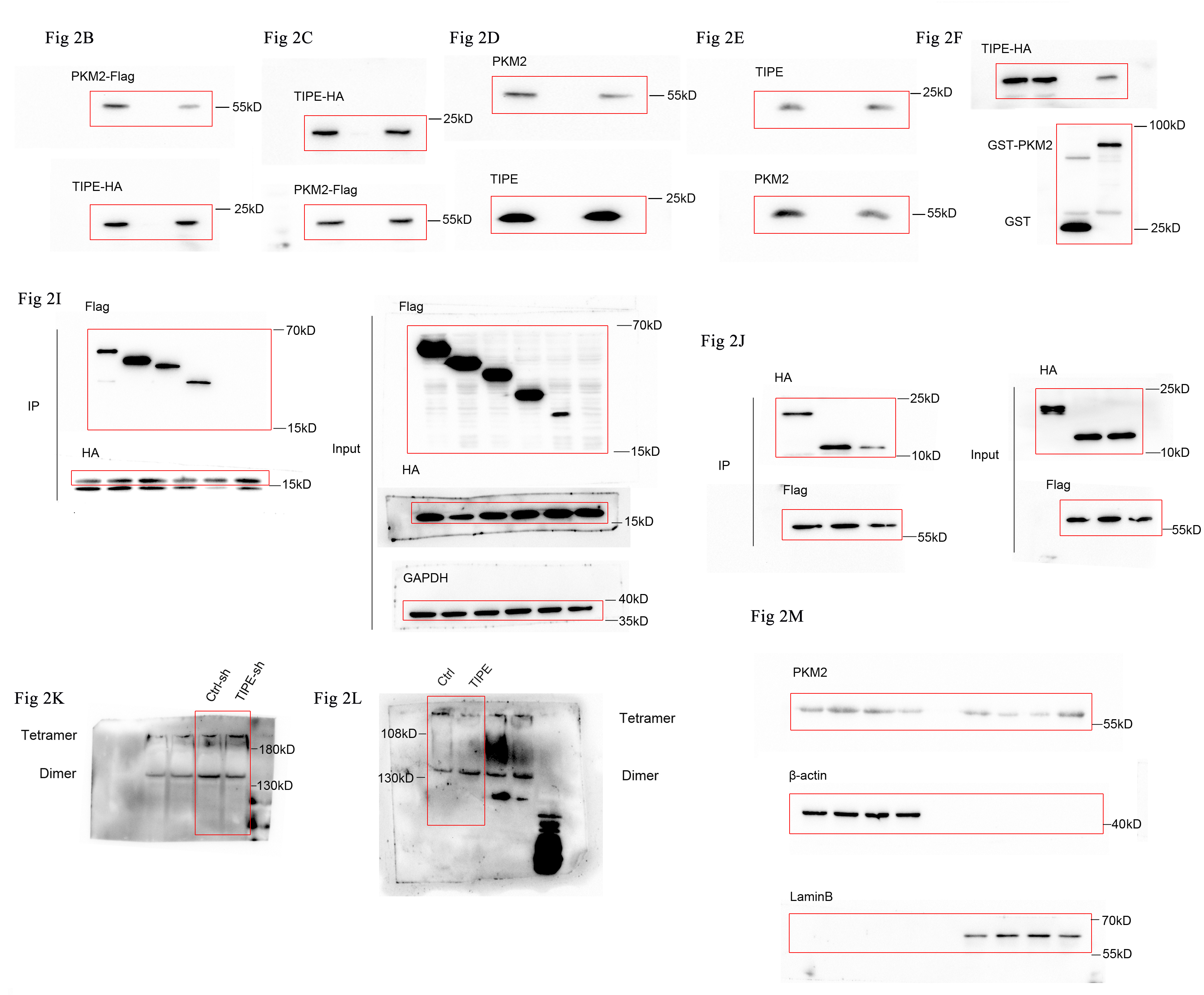

Supplement: Figure 2—source data 2. [file elife-92741-fig2-data2.zip › Figure 2–source data 2.tif]

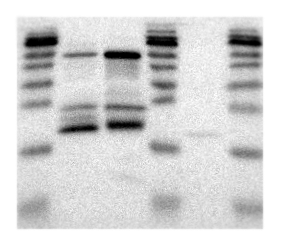

Supplement: Figure 2—figure supplement 1—source data 1. [file elife-92741-fig2-figsupp1-data1.zip › Figure 2-figure supplement 1-source data 2/5c.tif]

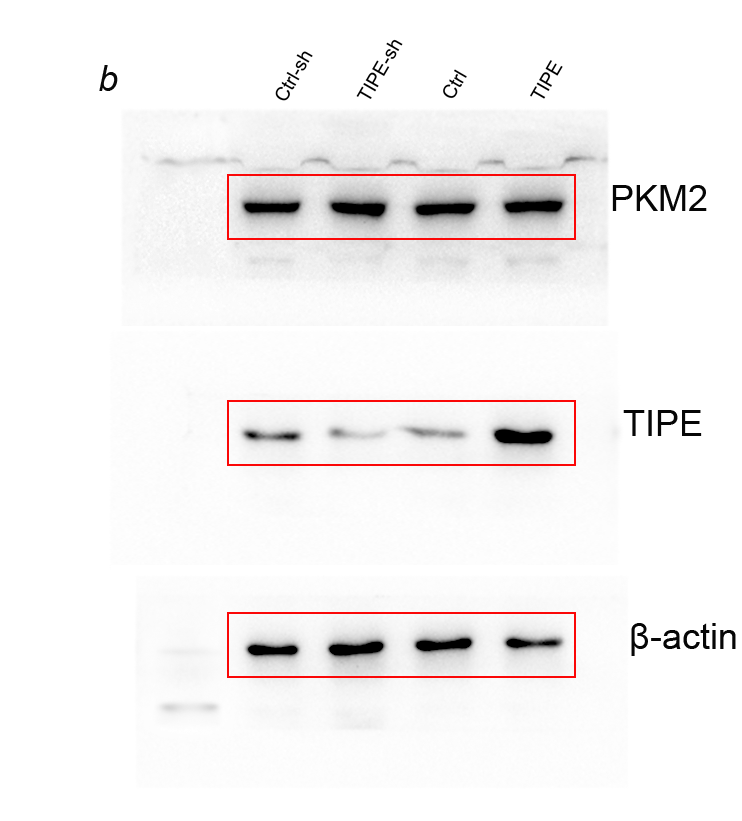

Supplement: Figure 2—figure supplement 1—source data 2. [file elife-92741-fig2-figsupp1-data2.zip › Figure 2-figure supplement 2-source data 2.tif]

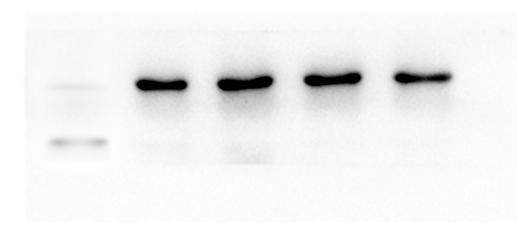

Supplement: Figure 2—figure supplement 2—source data 1. [file elife-92741-fig2-figsupp2-data1.zip › Figure 2-figure supplement 2-source data 2/6b actin.tif]

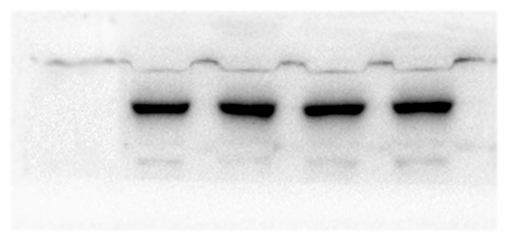

Supplement: Figure 2—figure supplement 2—source data 1. [file elife-92741-fig2-figsupp2-data1.zip › Figure 2-figure supplement 2-source data 2/6b PKM2.tif]

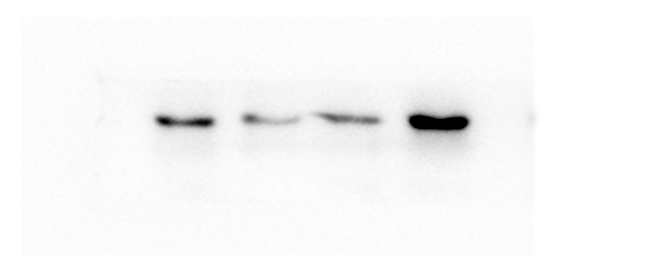

Supplement: Figure 2—figure supplement 2—source data 1. [file elife-92741-fig2-figsupp2-data1.zip › Figure 2-figure supplement 2-source data 2/6b TIPE.tif]

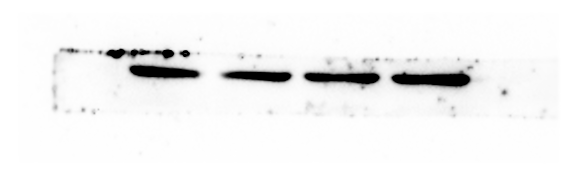

Supplement: Figure 3—source data 1. [file elife-92741-fig3-data1.zip › Figure 3-source data 2/Figure 3I GAPDH.tif]

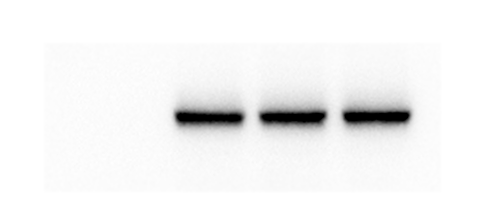

Supplement: Figure 3—source data 1. [file elife-92741-fig3-data1.zip › Figure 3-source data 2/Figure 3I Input-Flag.tif]

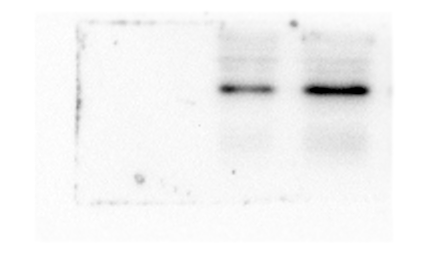

Supplement: Figure 3—source data 1. [file elife-92741-fig3-data1.zip › Figure 3-source data 2/Figure 3I Input-HA.tif]

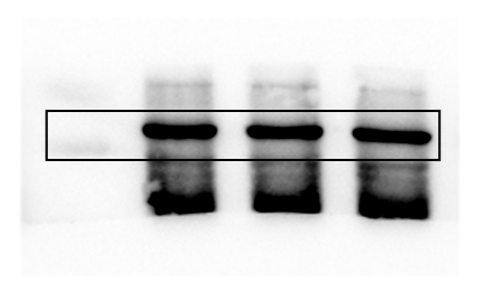

Supplement: Figure 3—source data 1. [file elife-92741-fig3-data1.zip › Figure 3-source data 2/Figure 3I Input-His.tif]

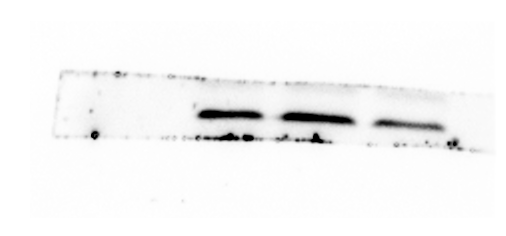

Supplement: Figure 3—source data 1. [file elife-92741-fig3-data1.zip › Figure 3-source data 2/Figure 3I IP-Flag.tif]

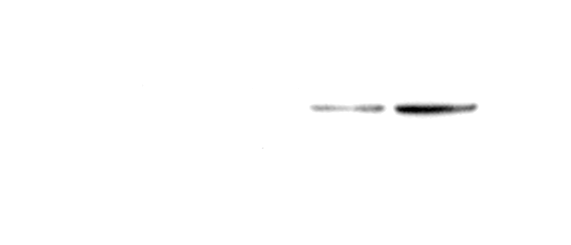

Supplement: Figure 3—source data 1. [file elife-92741-fig3-data1.zip › Figure 3-source data 2/Figure 3I IP-HA.tif]

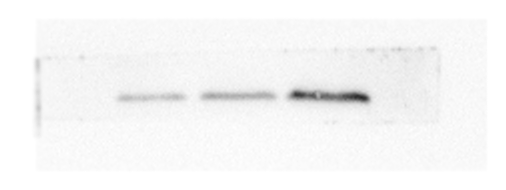

Supplement: Figure 3—source data 1. [file elife-92741-fig3-data1.zip › Figure 3-source data 2/Figure 3I IP-His.tif]

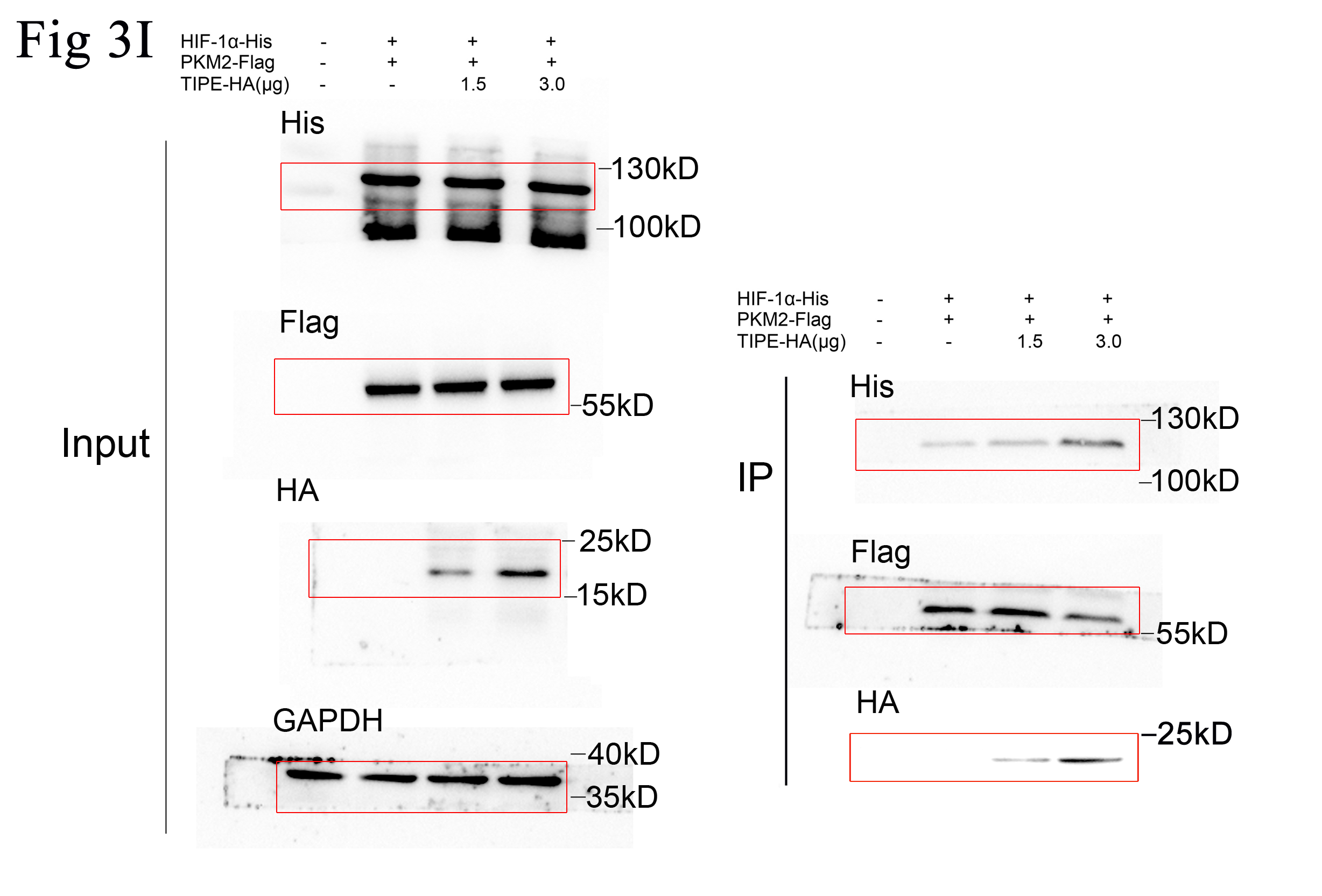

Supplement: Figure 3—source data 2. [file elife-92741-fig3-data2.zip › Figure 3–source data 2.tif]

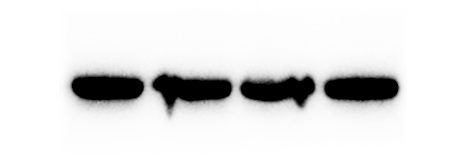

Supplement: Figure 4—source data 1. [file elife-92741-fig4-data1.zip › Figure 4-source data 2/Figure 4A GAPDH.tif]

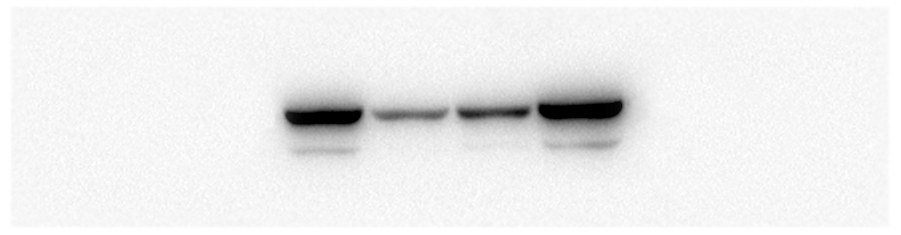

Supplement: Figure 4—source data 1. [file elife-92741-fig4-data1.zip › Figure 4-source data 2/Figure 4A p-PKM2(Ser37).tif]

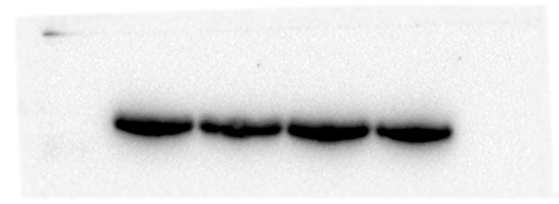

Supplement: Figure 4—source data 1. [file elife-92741-fig4-data1.zip › Figure 4-source data 2/Figure 4A p-PKM2(Tyr105).tif]

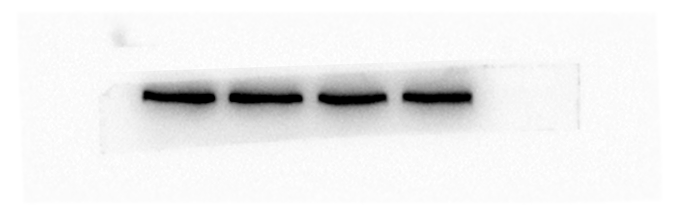

Supplement: Figure 4—source data 1. [file elife-92741-fig4-data1.zip › Figure 4-source data 2/Figure 4A PKM2.tif]

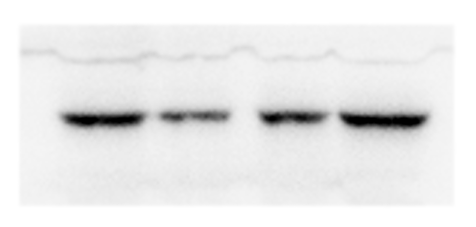

Supplement: Figure 4—source data 1. [file elife-92741-fig4-data1.zip › Figure 4-source data 2/Figure 4A TIPE.tif]

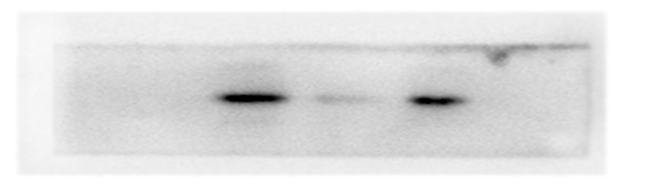

Supplement: Figure 4—source data 1. [file elife-92741-fig4-data1.zip › Figure 4-source data 2/Figure 4B Flag.tif]

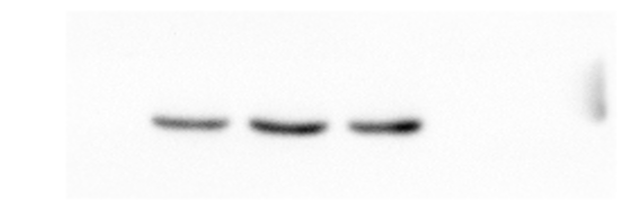

Supplement: Figure 4—source data 1. [file elife-92741-fig4-data1.zip › Figure 4-source data 2/Figure 4B GAPDH.tif]

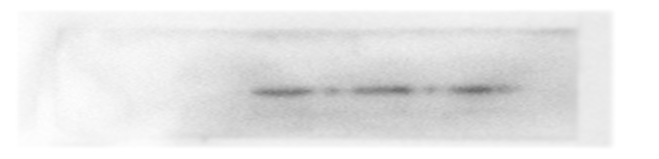

Supplement: Figure 4—source data 1. [file elife-92741-fig4-data1.zip › Figure 4-source data 2/Figure 4B HA.tif]

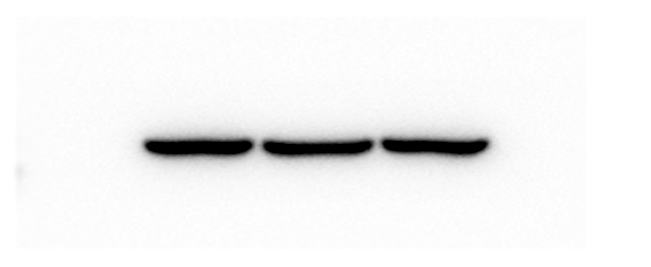

Supplement: Figure 4—source data 1. [file elife-92741-fig4-data1.zip › Figure 4-source data 2/Figure 4B Input-Flag.tif]

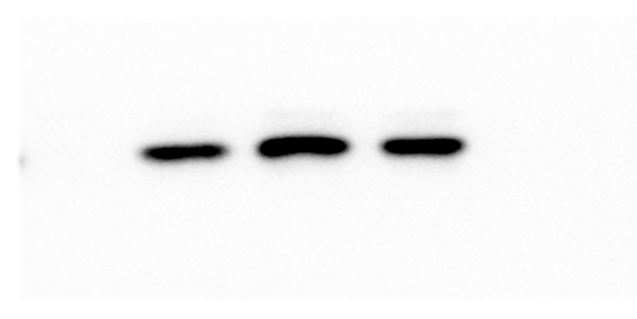

Supplement: Figure 4—source data 1. [file elife-92741-fig4-data1.zip › Figure 4-source data 2/Figure 4B Input-HA.tif]

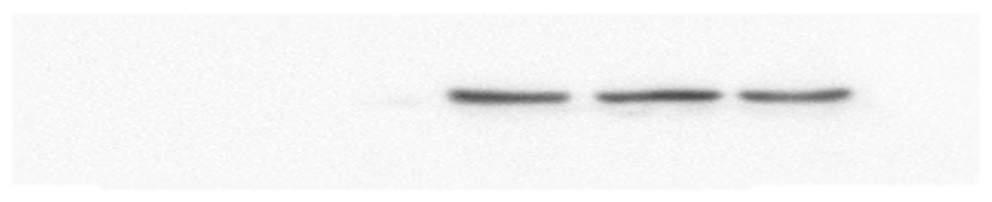

Supplement: Figure 4—source data 1. [file elife-92741-fig4-data1.zip › Figure 4-source data 2/Figure 4C Flag.tif]

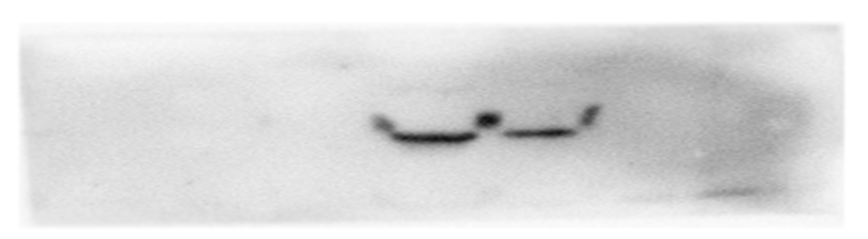

Supplement: Figure 4—source data 1. [file elife-92741-fig4-data1.zip › Figure 4-source data 2/Figure 4C HA.tif]

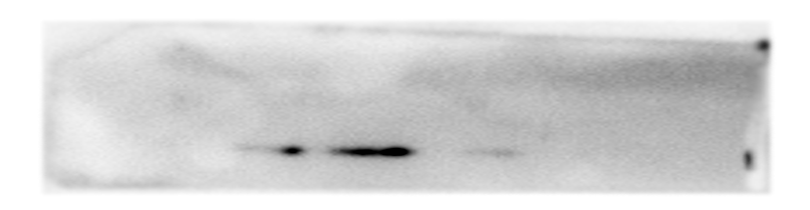

Supplement: Figure 4—source data 1. [file elife-92741-fig4-data1.zip › Figure 4-source data 2/Figure 4C His.tif]

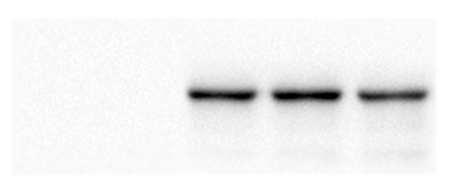

Supplement: Figure 4—source data 1. [file elife-92741-fig4-data1.zip › Figure 4-source data 2/Figure 4C Input Flag.tif]

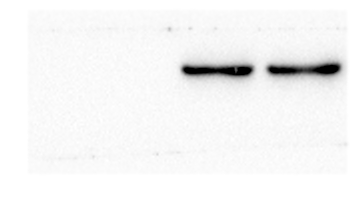

Supplement: Figure 4—source data 1. [file elife-92741-fig4-data1.zip › Figure 4-source data 2/Figure 4C Input HA.tif]

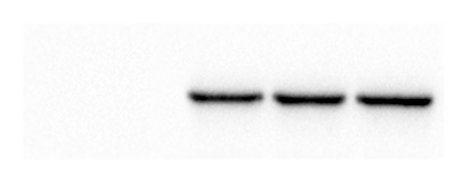

Supplement: Figure 4—source data 1. [file elife-92741-fig4-data1.zip › Figure 4-source data 2/Figure 4C Input His.tif]

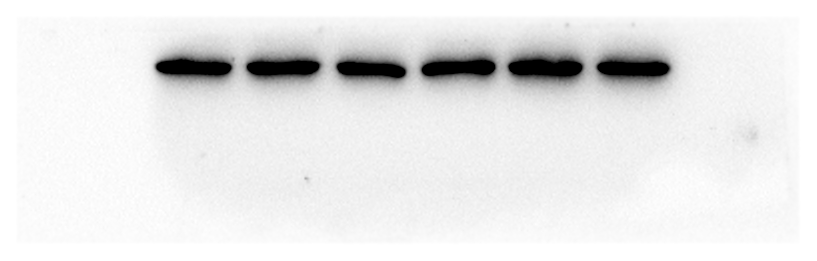

Supplement: Figure 4—source data 1. [file elife-92741-fig4-data1.zip › Figure 4-source data 2/Figure 4D actin.tif]

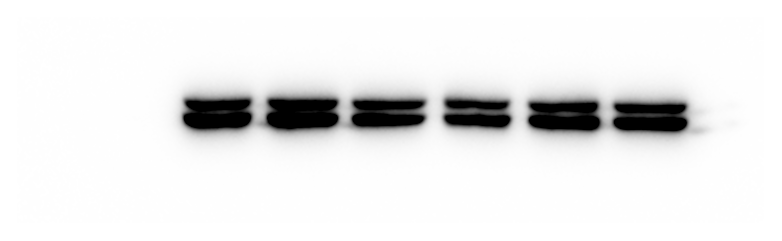

Supplement: Figure 4—source data 1. [file elife-92741-fig4-data1.zip › Figure 4-source data 2/Figure 4D ERK.tif]

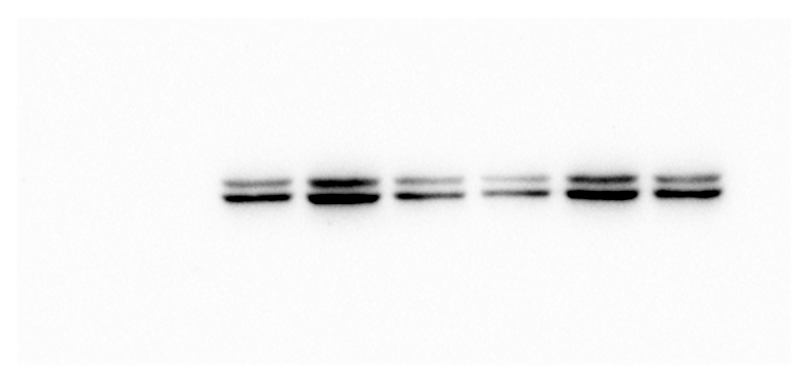

Supplement: Figure 4—source data 1. [file elife-92741-fig4-data1.zip › Figure 4-source data 2/Figure 4D p-ERK.tif]

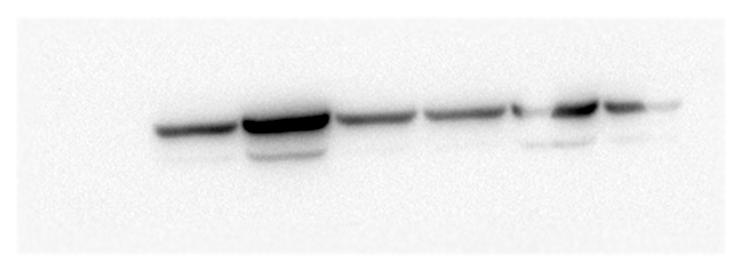

Supplement: Figure 4—source data 1. [file elife-92741-fig4-data1.zip › Figure 4-source data 2/Figure 4D p-PKM2(Ser37).tif]

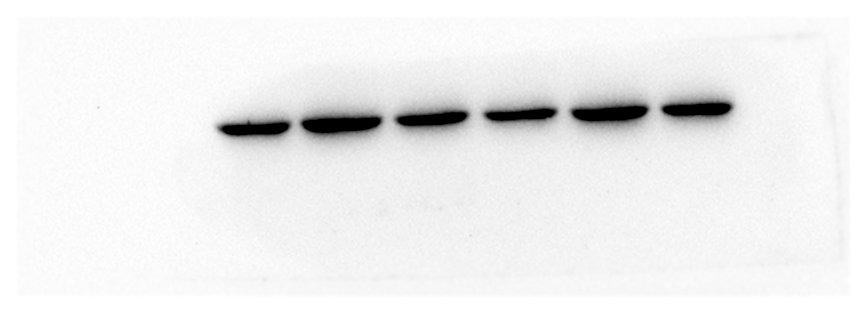

Supplement: Figure 4—source data 1. [file elife-92741-fig4-data1.zip › Figure 4-source data 2/Figure 4D PKM2.tif]

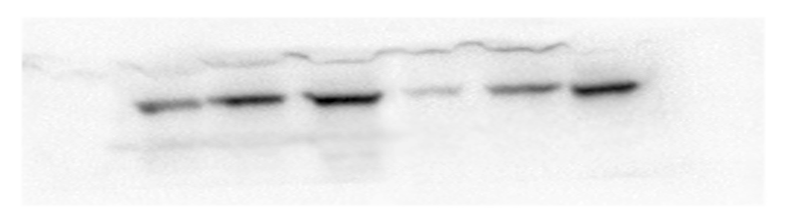

Supplement: Figure 4—source data 1. [file elife-92741-fig4-data1.zip › Figure 4-source data 2/Figure 4D TIPE-2.tif]

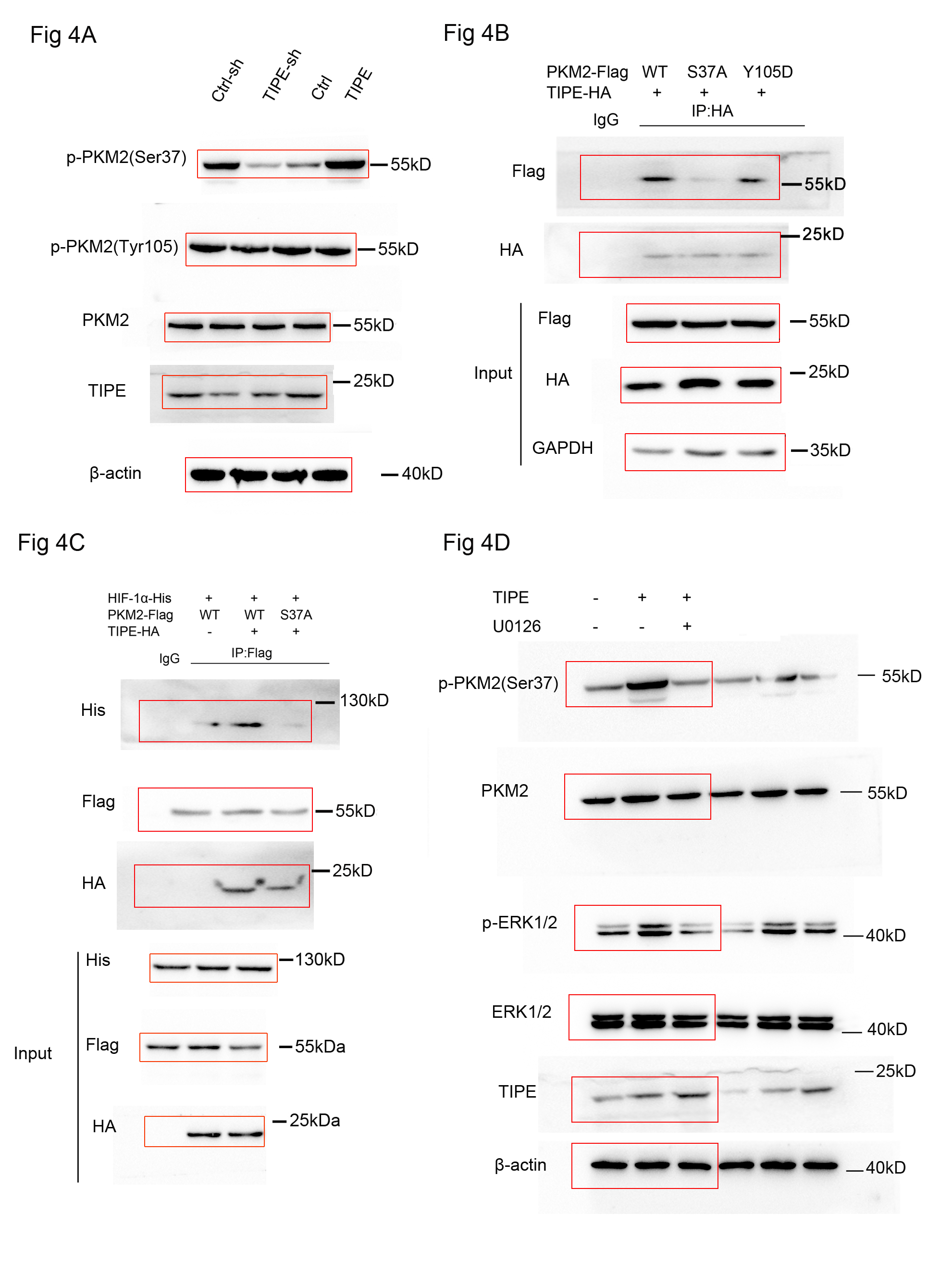

Supplement: Figure 4—source data 2. [file elife-92741-fig4-data2.zip › Figure 4–source data 2.tif]
