# Supplementary material for: TIPE drives a cancer stem-like phenotype by promoting glycolysis via PKM2/HIF-1α axis in melanoma
Source: eLife. 2024 Dec 27;13:RP92741. doi: 10.7554/eLife.92741 (PMC11677236; doi:10.7554/eLife.92741)
Supplement: Supplementary file 1. — (a) The top candidate interacting proteins of TIPE identified by mass spectrometry. (b) Genes upregulated in response to low-oxygen levels (hypoxia) in melanoma. (c) Limiting dilution data. (d) Confidence intervals for 1/(stem cell frequency). (e) Primer or siRNA sequences. (f) Experimental materials. (g) The clinicopathological characteristics of 48 melanoma specimens. [file elife-92741-supp1.docx]

**supplementary file 1a**

**The top candidate interacting proteins of TIPE identified by mass spectrometry**

| **Accession** | **Protein name** | **Gene name** | **Coverage(%)** | **#Peptides** |
| --- | --- | --- | --- | --- |
| P35527 | Keratin type I cytoskeletal 9 | KRT9 | 54 | 29 |
| P60709 | Actin cytoplasmic 1 | ACTB | 45 | 12 |
| P02751 | Fibronectin | FN1 | 11 | 22 |
| P68363 | Tubulin alpha-1B chain | TUBA1B | 37 | 10 |
| Q9BQE3 | Tubulin alpha-1C chain | TUBA1C | 33 | 9 |
| P13647 | Keratin type II cytoskeletal 5 | KRT5 | 34 | 24 |
| P35579 | Myosin-9 | MYH9 | 6 | 6 |
| P02533 | Keratin type I cytoskeletal 14 | KRT14 | 36 | 15 |
| P21333 | Filamin-A | FLNA | 5 | 6 |
| O60832 | H/ACA ribonucleoprotein complex subunit DKC1 | DKC1 | 17 | 11 |
| P63267 | Actin gamma-enteric smooth muscle | ACTG2 | 16 | 5 |
| P68032 | Actin alpha cardiac muscle 1 | ACTC1 | 16 | 5 |
| P68133 | Actin alpha skeletal muscle | ACTA1 | 16 | 5 |
| P62736 | Actin aortic smooth muscle | ACTA2 | 16 | 5 |
| Q14498 | RNA-binding protein 39 | RBM39 | 17 | 7 |
| P11387 | DNA topoisomerase 1 | TOP1 | 12 | 8 |
| P09211 | Glutathione S-transferase P | GSTP1 | 29 | 4 |
| P05787 | Keratin type II cytoskeletal 8 | KRT8 | 23 | 14 |
| P08779 | Keratin type I cytoskeletal 16 | KRT16 | 19 | 10 |
| **P14618** | **Pyruvate kinase PKM** | **PKM** | **19** | **5** |
| Q7Z794 | Keratin type II cytoskeletal 1b | KRT77 | 10 | 9 |
| P13646 | Keratin type I cytoskeletal 13 | KRT13 | 10 | 6 |

**supplementary file 1b**

**Genes up-regulated in response to low oxygen levels (hypoxia) in melanoma**

| **Hypoxia score was calculated by single sample GSEA (ssGSEA)** | | | | |
| --- | --- | --- | --- | --- |
| \| *ACKR3* \| \| --- \| \| *ADM* \| \| *ADORA2B* \| \| *AK4* \| \| *AKAP12* \| \| *ALDOA* \| \| *ALDOB* \| \| *ALDOC* \| \| *AMPD3* \| \| *ANGPTL4* \| \| *ANKZF1* \| \| *ANXA2* \| \| *ATF3* \| \| *ATP7A* \| \| *B3GALT6* \| \| *B4GALNT2* \| \| *BCAN* \| \| *BCL2* \| \| *BGN* \| \| *BHLHE40* \| \| *BNIP3L* \| \| *BRS3* \| \| *BTG1* \| \| *CA12* \| \| *CASP6* \| \| *CAV1* \| \| *CAVIN1* \| \| *CAVIN3* \| \| *CCN1* \| \| *CCN2* \| \| *CCN5* \| \| *CCNG2* \| \| *CDKN1A* \| \| *CDKN1B* \| \| *CDKN1C* \| \| *CHST2* \| \| *CHST3* \| \| *CITED2* \| \| *COL5A1* \| \| *CP* \| | \| *CSRP2* \| \| --- \| \| *CXCR4* \| \| *DCN* \| \| *DDIT3* \| \| *DDIT4* \| \| *DPYSL4* \| \| *DTNA* \| \| *DUSP1* \| \| *EDN2* \| \| *EFNA1* \| \| *EFNA3* \| \| *EGFR* \| \| *ENO1* \| \| *ENO2* \| \| *ENO3* \| \| *ERO1A* \| \| *ERRFI1* \| \| *ETS1* \| \| *EXT1* \| \| *F3* \| \| *FAM162A* \| \| *FBP1* \| \| *FOS* \| \| *FOSL2* \| \| *FOXO3* \| \| *GAA* \| \| *GALK1* \| \| *GAPDH* \| \| *GAPDHS* \| \| *GBE1* \| \| *GCK* \| \| *GCNT2* \| \| *GLRX* \| \| *GPC1* \| \| *GPC3* \| \| *GPC4* \| \| *GPI* \| \| *GRHPR* \| \| *GYS1* \| \| *HAS1* \| | \| *HDLBP* \| \| --- \| \| *HEXA* \| \| *HK1* \| \| *HK2* \| \| *HMOX1* \| \| *HOXB9* \| \| *HS3ST1* \| \| *HSPA5* \| \| *IDS* \| \| *IER3* \| \| *IGFBP1* \| \| *IGFBP3* \| \| *IL6* \| \| *ILVBL* \| \| *INHA* \| \| *IRS2* \| \| *ISG20* \| \| *JMJD6* \| \| *JUN* \| \| *KDELR3* \| \| *KDM3A* \| \| *KIF5A* \| \| *KLF6* \| \| *KLF7* \| \| *KLHL24* \| \| *LALBA* \| \| *LARGE1* \| \| *LDHA* \| \| *LDHC* \| \| *LOX* \| \| *LXN* \| \| *MAFF* \| \| *MAP3K1* \| \| *MIF* \| \| *MT1E* \| \| *MT2A* \| \| *MXI1* \| \| *MYH9* \| \| *NAGK* \| \| *NCAN* \| | \| *NDRG1* \| \| --- \| \| *NDST1* \| \| *NDST2* \| \| *NEDD4L* \| \| *NFIL3* \| \| *NOCT* \| \| *NR3C1* \| \| *P4HA1* \| \| *P4HA2* \| \| *PAM* \| \| *PCK1* \| \| *PDGFB* \| \| *PDK1* \| \| *PDK3* \| \| *PFKFB3* \| \| *PFKL* \| \| *PFKP* \| \| *PGAM2* \| \| *PGF* \| \| *PGK1* \| \| *PGM1* \| \| *PGM2* \| \| *PHKG1* \| \| *PIM1* \| \| *PKLR* \| \| *PKP1* \| \| *PLAC8* \| \| *PLAUR* \| \| *PLIN2* \| \| *PNRC1* \| \| *PPARGC1A* \| \| *PPFIA4* \| \| *PPP1R15A* \| \| *PPP1R3C* \| \| *PRDX5* \| \| *PRKCA* \| \| *PYGM* \| \| *RBPJ* \| \| *RORA* \| \| *RRAGD* \| | \| *S100A4* \| \| --- \| \| *SAP30* \| \| *SCARB1* \| \| *SDC2* \| \| *SDC3* \| \| *SDC4* \| \| *SELENBP1* \| \| *SERPINE1* \| \| *SIAH2* \| \| *SLC25A1* \| \| *SLC2A1* \| \| *SLC2A3* \| \| *SLC2A5* \| \| *SLC37A4* \| \| *SLC6A6* \| \| *SRPX* \| \| *STBD1* \| \| *STC1* \| \| *STC2* \| \| *SULT2B1* \| \| *TES* \| \| *TGFB3* \| \| *TGFBI* \| \| *TGM2* \| \| *TIPARP* \| \| *TKTL1* \| \| *TMEM45A* \| \| *TNFAIP3* \| \| *TPBG* \| \| *TPD52* \| \| *TPI1* \| \| *TPST2* \| \| *UGP2* \| \| *VEGFA* \| \| *VHL* \| \| *VLDLR* \| \| *WSB1* \| \| *XPNPEP1* \| \| *ZFP36* \| \| *ZNF292* \| |

**supplementary file 1c**

**Limiting dilution data**

| **A375** | **Cell number of injection** | **Ctrl-sh** | **TIPE-sh** |
| --- | --- | --- | --- |
|  | 10000 | 2/5 | 0/5 |
| **Frequency** | 100000 | 5/5 | 2/5 |
|  | 100000 | 5/5 | 5/5 |

**supplementary file 1d**

**Confidence intervals for 1/ (****stem cell frequency)**

| **A375** | **Lower** | **Estimate** | **Upper** | ***P* value** |
| --- | --- | --- | --- | --- |
| **Ctrl-sh** | 60864 | 18441 | 5588 | *P*<0.001 |
| **TIPE-sh** | 624917 | 202749 | 65780 |  |

**supplementary file 1e** **Primer or siRNA sequences**

| Primer sequences | |  |
| --- | --- | --- |
| *NANOG* | F: AATACCTCAGCCTCCAGCAGATG  R: TGCGTCACACCATTGCTATTCTTC | |
| *NOTCH1* | F: CCTGAGGGCTTCAAAGTGTC  R: CGGAACTTCTTGGTCTCCAG | |
| *POU5F1* | F: CTTGCTGCAGAAGTGGGTGGAGGAA  R: CGGAACTTCTTGGTCTCCAG | |
| *SOX2* | F: AAATGGGAGGGGTGCAAAAGAGGAG  R: CAGCTGTCATTTGCTGTGGGTGATG | |
| *BMI-1* | F: TGGAGAAGGAATGGTCCACTTC  R: CAGCTGTCATTTGCTGTGGGTGATG | |
| *TIPE* | F: TTCAGGCCTCCCTCTTTAACAATC  R: CGTTCGTGGCAGGGGTTATT | |
| *HIF-1α* | F: CCAGTTAGGTTCCTTCGATCAGT  R: TTTGAGGACTTGCGCTTTCA | |
| *PKM2* | F: TTGCAGCTATTCGAGGAACTCCG  R: CACGATAATGGCCCCACTGC | |
| *18S rRNA* | F: CGGCTACCAC ATCCAAGGAA  R: GCTGGAATTACCGCGGCT | |
| *NES* | F: AGCTGGCGCACCTCAAGATGT  R: CCTGAAAGCTGAGGGAAGTCT | |
| *SOX10* | F: TCATGGTGTGGGCTCAGGCA  R: CGCTTGTCACTTTCGTTCAGC | |
| *LDHA(HRE)* | F: TTGGAGGGCAGCACCTTACTTAGA  R: GCCTTAAGTGGAACAGCTATGCTGAC | |
| *SLC2A1(HRE)* | F: CTGTAATCCCAGCTACTCGG  R: CACGATCTCGGCTCACTGTA | |
| *LDHA* | F: ATCTTGACCTACGTGGCTTGGA  R: CCATACAGGCACACTGGAATCTC | |
| *SLC2A1* | F: CGGGCCAAGAGTGTGCTAAA  R: TGACGATACCGGAGCCAATG | |
| siRNA/shRNA sequence | |  |
| TIPE-sh1 | gtTTCCATCAGGTGGATTATA | |
| TIPE-sh2 | ccACCTTAATAGACGACACAA | |
| PKM2-sh | Purchased from Santa Cruz Biotechnology, Inc. (#sc-62820-SH) | |

**supplementary file 1f**

**Experimental materials**

| Reagent or resource | Source | Identifier |
| --- | --- | --- |
| Antibodies for western blot or immunofluorescence staining | | |
| TIPE | Abcam | ab195810 |
| p-PKM2(Ser37) | Affinity | DF7772 |
| PKM2(Rabbit) | Bioworld | BS6443 |
| PKM2(Mouse) | Invitrogen | MA5-32976 |
| p-PKM2(Tyr105) | Affinity | DF2975 |
| Flag | Bioworld | AP0007 |
| HA | Bioworld | AP0005M |
| His | Zenbio | 350175 |
| CD44, APC | Multisciences | AH04405 |
| LDH | Bioworld | MB63796 |
| HIF-1α(Mouse) | Cloud-Clone | MAA798Hu22 |
| HIF-1α(Rabbit) | Abcam | ab179483 |
| ERK1/2 | CST | #5013 |
| p-ERK1/2 | CST | #4370 |
| Lamin B1 | Proteintech | 12987-1-AP |

**supplementary file 1g**

**The** **clinicopathological characteristics of 48 melanoma specimens**

| **Parameters** | **Number of cases (%)** |
| --- | --- |
| **Gender** |  |
| Female | 25 (52.08%) |
| Male | 23 (47.92%) |
| **Age (years)** |  |
| < 60 | 25 (52.08%) |
| ≥ 60 | 23 (47.92%) |
| **Pathological type** |  |
| Malignant melanoma | 48 (100.00%) |
| **T classification** |  |
| T3 | 4 (8.33%) |
| T4 | 28 (58.33%) |
| Data missing | 16(33.33%) |
| **N classification** |  |
| N0 | 22 (45.83%) |
| N1 | 10 (20.83%) |
| Data missing | 16(33.33%) |
| **Clinical stage** |  |
| II | 27 (56.25%) |
| III | 1 (2.08%) |
| IV | 4 (8.33%) |
| Data missing | 16(33.33%) |
